# Shiny-phyloseq: Web application for interactive microbiome analysis with provenance tracking

**DOI:** 10.1093/bioinformatics/btu616

**Published:** 2014-09-26

**Authors:** Paul J. McMurdie, Susan Holmes

**Affiliations:** Department of Statistics, Stanford University, Stanford, CA 94305, USA

## Abstract

**Summary:** We have created a Shiny-based Web application, called *Shiny-phyloseq*, for dynamic interaction with microbiome data that runs on any modern Web browser and requires no programming, increasing the accessibility and decreasing the entrance requirement to using phyloseq and related R tools. Along with a data- and context-aware dynamic interface for exploring the effects of parameter and method choices, Shiny-phyloseq also records the complete user input and subsequent graphical results of a user’s session, allowing the user to archive, share and reproduce the sequence of steps that created their result—without writing any new code themselves.

**Availability and implementation**: Shiny-phyloseq is implemented entirely in the R language. It can be hosted/launched by any system with R installed, including Windows, Mac OS and most Linux distributions. Information technology administrators can also host Shiny-phyloseq from a remote server, in which case users need only have a Web browser installed. Shiny-phyloseq is provided free of charge under a GPL-3 open-source license through GitHub at http://joey711.github.io/shiny-phyloseq/.

**Contact**: mcmurdie@alumni.stanford.edu.

## 1 INTRODUCTION

Analysis of microbial communities requires the interpretation of one or more high-dimensional abundance matrices and its relationship with other datasets, using a complex and emerging suite of methods from ecology, genetics, phylogenetics, multivariate statistics, visualization and testing. Filtering, custom curation and transformation of the abundance data are also required usually, but the precisely reproducible workflow from raw data to final analyses is often difficult or impossible to reproduce exactly. Ideally, published scientific analyses are completely reproducible in as easy a fashion as possible; and anything less represents an impediment to both progress and peer review (Peng, 2011). Fortunately, R is well suited for the analytical aspects of microbiome research, with an interaction-oriented functional programming design (R Development Core Team, 2014) that includes support for reproducible research (Allaire *et al.*, 2014; Xie, 2014). We recently described a software package for the R language, phyloseq, dedicated to the object-oriented representation and analysis of microbiome census data (McMurdie and Holmes, 2013). One of the originally stated goals of phyloseq was to leverage R-based resources for reproducible research, and thereby, improve the reproducibility and portability of published microbiome analyses. Unfortunately, for many microbiome researchers with classical training in biology, learning a programming language—even a functional interactive language like R—has proven to be a prohibitive investment of time and effort. However, most of the necessary computations are not only tractable by R, but also fast enough for dynamic interaction via a graphical user interface (GUI). Here we describe our release of ‘Shiny-phyloseq’, a Web browser GUI that leverages phyloseq and other R resources for the analysis of microbiome census data—while also allowing the user to archive the complete code and data necessary to exactly reproduce their session results. Although it is difficult for any single GUI application to support the full range of analyses required in microbiome research, Shiny-phyloseq provides a compelling framework and introduction to microbiome analysis in R. Its modular open-source design encourages modification, customization and code reuse.

There are other resources with GUI elements available for the analysis of microbiome census data, including MG-RAST (Meyer *et al.*, 2008), QIIME (Caporaso *et al.*, 2010) and CloVR (Angiuoli *et al.*, 2011). To our knowledge, there are no GUIs available for the analysis of microbiome census data that also leverage the R programming language, ggplot2 (Wickham, 2009), and phyloseq while also providing a ‘provenance record’ of a user’s session.

## 2 METHODS

Shiny-phyloseq is almost entirely R code, but, like any Shiny app, can be further customized/extended using HTML, CSS and JavaScript. Shiny-phyloseq is fully cross-platform and will launch locally from any R environment (Console R, Rgui, RStudio, etc.). It can also be hosted by a remote Web server—this latter case only requires that the user has a modern Web browser installed. Shiny-phyloseq leverages Shiny’s reactive programming framework to compartmentalize and cache expensive computational steps so that they are not recomputed unnecessarily during an interactive session.

The current implementation of Shiny-phyloseq is dependent on many important updates to the phyloseq package, including (i) an interface to DESeq2 (Anders and Huber, 2010) for a negative Binomial method recommended by McMurdie and Holmes, 2014; (ii) a ggplot2-friendly data organizing function, psmelt; (iii) the inclusion of low-level C code from APE (Paradis *et al.*, 2004) for ∼100× faster UniFrac (Hamady *et al.*, 2009) distance calculations and tree plotting; (iv) faster network plot function with better default settings, plot_net; and (v) additional options for ordering heatmaps by covariates.

## 3 BIOLOGICAL APPLICATIONS

A typical workflow in Shiny-phyloseq begins with data upload and selection, followed by optional filtering of Operational Taxonomic Units (OTUs) or samples. A number of exploratory data analysis methods are available in separate panels, including alpha diversity estimates, multivariate ordination methods, as well as network, heatmap, scatter and bar graphics. Some of these methods depend on data transformations (e.g. regularized log) or ecological distances (e.g. weighted UniFrac, Bray–Curtis), which can be selected from a sidebar panel of parameter-input widgets placed next to each graphic on each panel. Graphics can be downloaded in a user-specified format by clicking the download button at the bottom of each sidebar panel. Finally, a user can download a compressed file containing the complete code and data necessary to completely reproduce the steps that led to their graphical result.

Shiny-phyloseq provides new features, including (i) a context- and data-aware, browser-based interactive GUI application, (ii) interactive 3D network graphics based on d3.js, for exploring OTU or sample distance structure and (iii) provenance tracking for reproducible sessions.

**Fig. 1.**
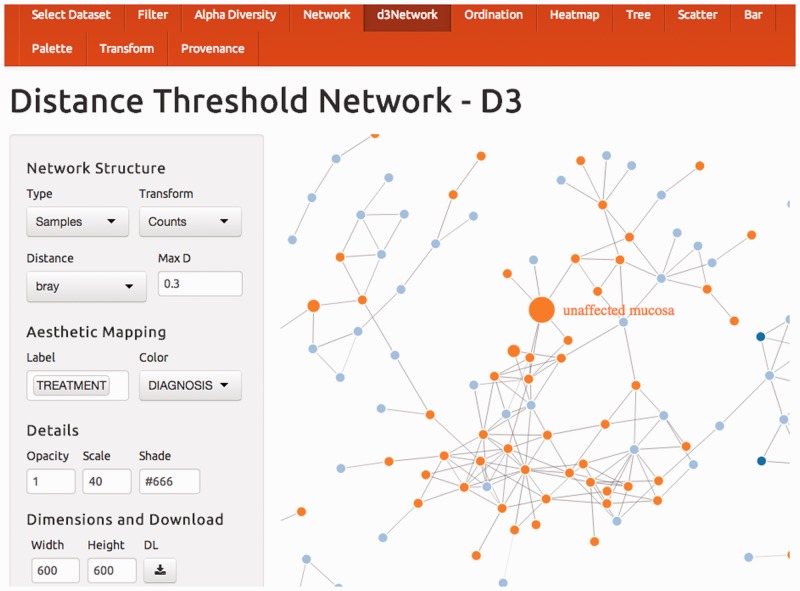
Shiny-phyloseq Network panel. The Shiny-phyloseq interface is organized into panels from left to right, beginning with data selection, filtering/curation, transformation and then graphic-specific panels. The user input widgets are consolidated in a left-hand sidebar of each panel. The *Select Dataset* panel begins each session. Pre-loaded datasets are available by default, and users can optionally specify public datasets hosted on QIIME-DB (Caporaso *et al.*, 2010), or upload private datasets in biom (McDonald *et al.*, 2012) or binary ‘.RData’ (phyloseq) formats. The *Filter* panel supports user-defined data filtering. Shiny-phyloseq provides a separate panel for each major graphic function in phyloseq, including alpha diversity, sample- or OTU-networks, barplot, heatmap, phylogenetic tree, ordination and scatterplot. All relevant panels support customization of figure dimensions, color palette and download file format

There are two network-graphic panels, both of which use new functionality. The *Network* panel features the ability to animate vertex connectedness as a change in the distance threshold, and scales edge thickness according to inverse distance value. The former feature can help a user to quickly scan the dependency of the network on the choice of global threshold. Alternatively, using the *d3Network* panel, networks can be interactively explored by dragging and stretching nodes in a live 3D network animation, with taxonomy or covariates mapped to node color and/or mouse-over labels (Fig. 1). This D3 (Bostock *et al.*, 2011) interactive graphic is a special phyloseq implementation of the d3Network package (Gandrud, 2014).

The final panel, *Provenance*, includes a button that (re)initiates a special processing of the Shiny event log into reusable R code. This record of parameter changes and analysis steps—when coupled with the complete workspace environment data—constitutes a so-called *provenance record* of the analysis session. Shiny-phyloseq initiates a browser download of the compressed file containing the code and data sufficient to exactly reproduce the series of graphics created in the session up to that point. This can be archived or shared for batch re-execution in R.
